# ToP: A Trend-of-Disease-Progression Procedure Works Well for Identifying Cancer Genes from Multi-State Cohort Gene Expression Data for Human Colorectal Cancer

**DOI:** 10.1371/journal.pone.0065683

**Published:** 2013-06-14

**Authors:** Feng-Hsiang Chung, Henry Hsin-Chung Lee, Hoong-Chien Lee

**Affiliations:** 1 Institute of Systems Biology and Bioinformatics, National Central University, Zhongli, Taiwan; 2 Department of Physics, Chung Yuan Christian University, Zhongli, Taiwan; 3 Center for Dynamical Biomarkers and Translational Medicine, National Central University, Zhongli, Taiwan; 4 Cathay Medical Research Institute, Cathay General Hospital, Taipei, Taiwan; Queen’s University Belfast, United Kingdom

## Abstract

Significantly expressed genes extracted from microarray gene expression data have proved very useful for identifying genetic biomarkers of diseases, including cancer. However, deriving a disease related inference from a list of differentially expressed genes has proven less than straightforward. In a systems disease such as cancer, how genes interact with each other should matter just as much as the level of gene expression. Here, in a novel approach, we used the network and disease progression properties of individual genes in state-specific gene-gene interaction networks (GGINs) to select cancer genes for human colorectal cancer (CRC) and obtain a much higher hit rate of known cancer genes when compared with methods not based on network theory. We constructed GGINs by integrating gene expression microarray data from multiple states – healthy control (Nor), adenoma (Ade), inflammatory bowel disease (IBD) and CRC – with protein-protein interaction database and Gene Ontology. We tracked changes in the network degrees and clustering coefficients of individual genes in the GGINs as the disease state changed from one to another. From these we inferred the state sequences Nor-Ade-CRC and Nor-IBD-CRC both exhibited a trend of (disease) progression (ToP) toward CRC, and devised a ToP procedure for selecting cancer genes for CRC. Of the 141 candidates selected using ToP, ∼50% had literature support as cancer genes, compared to hit rates of 20% to 30% for standard methods using only gene expression data. Among the 16 candidate cancer genes that encoded transcription factors, 13 were known to be tumorigenic and three were novel: CDK1, SNRPF, and ILF2. We identified 13 of the 141 predicted cancer genes as candidate markers for early detection of CRC, 11 and 2 at the Ade and IBD states, respectively.

## Introduction

Colorectal cancer (CRC) is the fourth leading cause of cancer death worldwide but rank higher in economically more developed societies. Like other types of cancer, CRC is a systems disease, a manifest of multiple functional disruptions in the tumorous cells. Global gene expression profiling using oligomeric DNA microarrays has been widely employed to gain insight in the underlying mechanisms for complex diseases, including CRC [1], [2]. Previous studies on gene expression profiles have provided distinct perspectives on the molecular etiology of CRC [3]–[6]. The overlap between published gene signatures from different studies for CRC tended to be small. Early on it was recognized the identification of differentially expressed genes (DEGs) in two cohort samples was a potentially useful approach [7]–[9]. Drawing an inference from a long list of DEGs is however a daunting task and may lead to widely varying results [10]. Gene sets analysis, a method based on *priori* biological information such as Gene Ontology (GO) and Kyoto Encyclopedia of Genes and Genomes (KEGG) on modules that are functionally annotated [10], partially meets the challenge. The rationale for this approach, which groups DEGs into functional subsets using GO or KEGG (or something equivalent), derives from the observation that most genes function as part of a group rather than singly [11]. However, because same-cohort genomic profiles are known to be highly heterogeneous, pre-grouped gene sets may not reflect the actual grouping in a cohort under study. Furthermore, a majority of human genes have not yet been assigned a definite pathway or protein complex [12].

Various causes of CRC have been revealed, but the global landscape for dynamic features of carcinogenesis processes remains unclear. Protein-protein interactions (PPIs) are fundamental to biological processes, and protein interaction networks (PINs) provide a global yet static view of cellular mechanisms in cell. Dynamic features of PINs may be uncovered through the integration of PPI data gene expression profiles [13]. Genes with correlated expression levels over different physiological states or over individuals in a cohort are likely to be involved in similar functions or cellular processes. For instance, genes regulated by a common transcription factor are expected to have correlated gene expressions. A gene interaction network (GGIN) constructed by integrating gene expression data with PPI data is meant to an interaction map of bio-molecules that indicate co-regulatory relationships, co-expression associations, downstream physical interaction between proteins encoded by the “interacting” genes, and possibly other relationships between genes [14]. Many methods employing, for instance, correlation coefficient [15], [16], mutual information [17], [18], simulated annealing [19], and reverse engineering approaches [20], [21] have been applied to re-construct GGINs for large-scale gene expression data in model organisms, including yeast and human. Several studies demonstrated the extraction of dynamic properties of condition-specific networks by integrating gene co-expression patterns and physical protein interactions [13], [22], [23].

With cancer being a systems disease, systemic changes in a cancerous cell during cancer progression are expected to measurably manifest in changes occurring in the GGINs constructed from data taken at different states of the disease. An important cause of cancer is serially accumulated gene mutations [24]. Recent systematic screenings of cancer genomes have revealed a significant number of functionally heterogeneous genes, or hubs, that are mutated in colorectal tumors [25]–[27]. Because hub genes are important in the function of a cell, we assumed that a change in the status of a hub gene had a higher probability than an average gene in reflecting an interrupted functional change in the cell. Thus, a hub gene in a normal state that became a non-hub gene should have a higher probability in reflecting a disease-linked loss in cell function, while the opposite may reflect a gain in cell function.

Here, we constructed GGINs for the four physiological states – normal (Nor), colorectal adenoma (Ade), inflammatory bowel disease (IBD), and CRC – by integrating gene expression data from four corresponding sets of cohort microarrays with Human Protein Reference Database (HPRD) [28]. In a given state, two genes were assumed to “interact” if there expression intensities were highly correlated and if proteins encoded by the pair were known to interact. Using the GGINs we constructed, we devised ToP (trend of progression) procedure, whereby genes whose degrees and clustering coefficients [29] in GGINs changed in step with the trend of the progression of cancer, or, genes that are not hubs in the Nor network but become hubs in the CRC network, were selected as potentially cancer genes.

We applied the ToP procedure to the state sequences Nor-Ade-CRC and Nor-IBD-CRC and selected genes with statistical significance (permutation test *p*-value <0.001) similar to those obtained by conventional methods as eBayes and SAM. However, genes selected by ToP had a much higher hit rate (∼50%, *p*-value <0.001) of known cancer genes than hit rates obtained by eBayes and SAM (∼20%, *p*-value ∼ 0.5). Because ToP based its analysis on data from a sequence of states, we also used it to identify potential biomarkers for early diagnostic detection of CRC at the Ade and at the IBD states.

## Materials and Methods

### Samples and Microarrays

Data provided by the Gyorffy group [30] on genome-wide gene expression profile from tissue samples of 53 human patients evaluated by HG-U133 Plus 2.0 platform microarrays (Affymetrix, Santa Clara), which list 18,267 genes, were downloaded from Gene Expression Omnibus (GEO) database (GEO accession no. GSE4183). The arrays were made from patients’ tissues grouped into four physiological states of frozen colonic biopsy: 8 for Nor, and 15 each for Ade, IBD, and CRC, respectively. Colon biopsies were taken during routine endoscopical intervention before treatment [31]. The accuracy of the microarray expression values were validated by TaqMan RT-PCR assay [30]. Analyses of microarray data carried out in this work were conducted in R environment (version 2.12.0).

### Selection of Significant DEGs

Significantly expressed genes were selected using the Significance Analysis of Microarrays algorithm (SAM) [9] and one-way analysis of variance (ANOVA) [32]. The statistical thresholds for the *p*-value of Student’s *t*-test and fold change used in SAM were determined using published real-time PCR results on 84 genes [30] (Figure S1). We used two modes, (1) the two-class unpaired mode for selecting genes whose mean expression level was significantly different in two groups of samples (analogous to between subjects *t*-test) and (2) the multi-class mode to select genes whose mean expression was different across a set of samples greater than two (analogous to one-way ANOVA). The empirical Bayes statistics (eBayes) was used as an alternative statistical model. For a review of these algorithms see in [33]. FDRs [34] were computed using both Student’s *t*-tests and ANOVA tests using random permutation in SAM through the R package “siggenes”.

### Construction of GGIN

Protein-protein interaction (PPI) information on 30,047 protein entries and 39,194 interactions was downloaded from HPRD [28] and were integrated with state specific microarray gene expression data to construct GGINs, one for each state. For a given state and a Pearson *p*-value (see below) threshold *p*_0_, we included a pair of genes in the GGIN if: (1) the *p*-value for the pair was not greater than *p*_0_; (2) the protein pair encoded by the gene pair was linked in the PPI data. For a given state and a set of microarray data, a Pearson’s correlation coefficient (PCC) between each gene-pair was calculated based on the intensities across the set for the pair. That is, if a set of *n* microarrays is used for the computation, the PCC is that between two sets of *n* intensities. Statistical inference based on PCC was performed by permutation tests and *t*-statistics. We call a *p*-value corresponding to a PPC a Pearson *p*-value. Network properties are *n*-dependent. Results given are for 8-sample networks. For the 8-sample Nor, one network was constructed (for each *p*_0_). For each of the other three 15-sample states, 100 networks were constructed, each from an eight-sample sets randomly selected from the 15 samples. We use standard network terminology. We say a node *i* with degree *k_i_* has *k_i_* neighbors. The clustering coefficient *C* of a node is the ratio of the number of links *e* among the neighbors of degree-*k* node to the number of possible such links: *C* = 2*e*/(*k*(*k*−1)) [29]. Layouts for networks were made using the open source platform Cytoscape (version 2.7.0) through the “edge-weighted spring-embedded” layout function. Default parameters values were used, except that the “number of iterations” for each node was increased to 200, and “strength” was changed to 1500 to avoid collisions. The plug-in “GOlorize” [35] was used to automatically assign colors to gene nodes to highlight enriched gene-ontology terms. The color and width of an edge were used to indicate sign and strength of correlation, respectively; red (blue) for positive (negative) correlation.

### Functional Sub-networks and FFN

Genes in each state-specific GGIN were assigned to over-represented biological functions as defined in GO term association [36]. Enrichment analyses based on conditional hypergeometric test [37] were made using the R package GOstats [38] downloaded from the Bioconductor website [39]. Based on functional gene sets a GGIIN was reduced to FFN for easier visual inspection.

### ToP and ToP+SAM (TPS) Procedures for Selecting Cancer Gene Discovery

**Figure 1:**
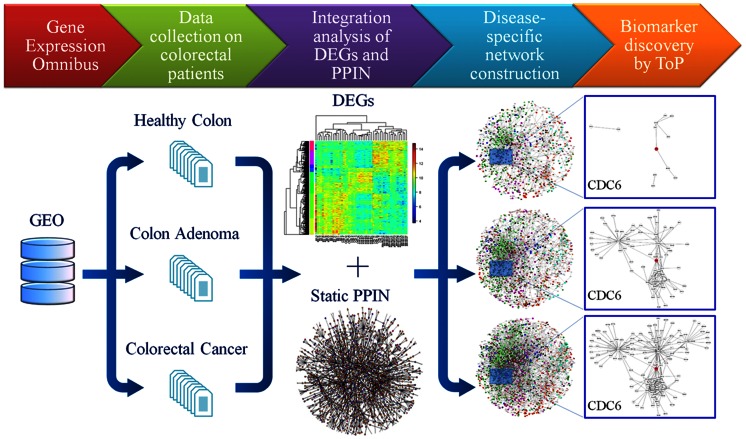
ToP procedure flow chart for selecting of CRC cancer genes. DEG, differentially expressed gene; PPIN, protein-protein interaction network. Boxes in the right-most column illustrate how the predicted tumorigenic gene CDC6 satisfies the ToP criteria: the gene-gene interaction sub-network associated with it grows markedly as the state progress from normal through adenoma to CRC.

The ToP procedure (Figure 1) applied to the sequence Nor-X-CRC (X = Ade or IBD, as the case may be) consisted of the steps: (1) Construct GGINs for Nor, X, and CRC using a threshold Pearson *p*-value <0.01. (2) Select a gene if: (a) it appears in at least one GGIN; (b) it at least in one GGIN satisfies degree *D* >4 and clustering coefficient *C* >0; (c) its *D* and *C* increase along the sequence (but no limitation is set on the Nor-X pair). (3) Form a separate category for predicted cancer genes encoding key transcription factors. In the TPS procedure, an extra filtering step added: (4) Limit the selected genes to be a DEG (adjusted *p*-values <0.05, fold change >1.5 or <1/1.5) at least in X vs. Nor or in CRC vs. Nor.

### Hit Rate for Cancer Genes

Hit rate was defined as the ratio of selected genes appearing given as a cancer-related gene in *CancerGenes* [40] to the total number of selected genes, given as a percentage. *CancerGenes* lists expert-annotated cancer related genes from key public databases including Cellmap.org (http://cancer.cellmap.org), Entrez Gene [41], and Sanger CGC [42], and cancer reviews [24], [42]–[44]. Total 3,165 genes were collected and various types of sources (e.g., cancer gene, tumor suppressor, stability gene, *etc.*) were all included in the hit rate calculation. Because the Affymetrix HG-U133 Plus 2.0 array platform lists 18,267 genes and *CancerGenes* lists 3,165 genes, a random selection of genes would yield a hit rate close to 20%.

### Randomization

We performed two kinds of randomizations. Type-1: Separately for every gene, scramble the intensities on entire set of arrays. In each case of randomization, one sweep over all the genes was performed. This process conserves the distribution of intensities for each gene but destroys the intensity correlation between gene pairs. Type-2: randomly assign gene pairs to each link in a network. The procedure conserved the number of links but not the topology of a network. In each randomization, one sweep over all the links in the network was performed. This process conserves the number of links in, but not the topology of, the network. We tried a third, type-3, topology-conserving randomization on networks, in which the topology was left unchanged but genes were randomly assigned to nodes in a network. This proved to be not a true randomization.

### Selection of Markers for Early Diagnostic Detection of CRC

Biomarkers for early detection in the Ade state were selected from the TPS gene set for the Nor-Ade-CRC sequence (see Results) those having a five-fold or more increase in (network) degree from Nor to Ade and being a DEG with a *p*-value <0.0001 in Ade vs. Nor. Similarly for biomarkers for early detection in the IBD state, with IBD replacing Ade.

## Results

### Significant Differentially Expressed Genes

The total set of selected 2,666 DEGs (FDR <0.001, Student’s *t*-test (in SAM) *p*-value <0.05, fold change >1.5; Figure S1) was the union DEGs separately selected from three state pairs; ADE vs. NOR, 1652 genes; CRC vs. NOR, 1100 genes; IBD vs. NOR: 1629 genes. The DEGs were classified according to GO into eleven functional modules: DNA replication, DNA repair, cell cycle, cell proliferation, RNA metabolism, transcription, translation, apoptosis, signal transduction, immune system, cell adhesion (Table S1). A heat map generated by the two-way unsupervised hierarchical clustering method (Figure S2) shows the fragmentation into two parts of CRC, reflecting relative heterogeneity in the cancer samples. However, no difficulty in extracting CRC specific DEGs was encountered.

### Disease Networks were Larger and more Complex, and CRC Network had Highest Complexity

**Figure 2:**
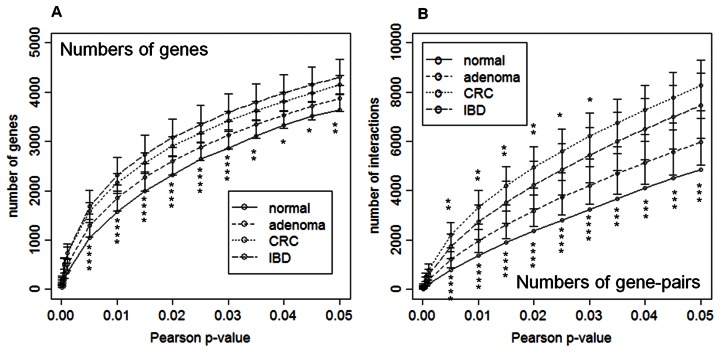
Number of genes and gene-pair interactions in networks as functions of Pearson p-value. Number of genes (A) and gene-pair interactions (B) in the disease specific networks, as functions of Pearson *p*-value threshold, *p*_0_, in the 8-sample gene-networks of patients belonging to the four state-types: Nor, Ade, IBD, and CRC. Non-Nor results are averaged over 100 random 8-sample sets. Error bars indicate standard deviations. Asterisks above (below) the curves give *p*-values of two-sample Student’s *t*-test between CRC and IBD (CRC and Nor): * *p*-value<10^−4^; ** *p*-value<10^−8^; *** *p*-value<10^−12^; **** *p*-value<10^−16^.

Results for GGINs given are for 8-sample networks. There was one GGIN but 100 GGINs for each of the disease states were constructed (see Methods). The number of genes and (gene-gene) links both decreased with decreasing Pearson *p*-value threshold *p*_0_ [45] in constructed GGINs (Figure 2), as expected. For given *p*_0_ both the gene and link numbers increased in the progression Nor to Ade to IBD/CRC. Gene number in the IBD network was slightly larger than in CRC, but the link number in CRC was significantly larger than IBD. The degree distributions of the four networks obeyed power-laws. In terms of network complexity (Table 1), the four networks belongs to three groups, in ascending order of complexity: Nor, Ade and IBD, and CRC. All four networks were composed of connected sub-networks, or clusters. The three disease networks were each dominated by a giant cluster, containing (on average) 760, 971, and 1388 genes, for Ade, IBD, and CRC, respectively. The Nor network does not have a giant cluster; its two largest clusters respectively had 219 and 73 genes.

**Table 1:** Structural parameters for the four gene-gene interaction networks* (GGINs).

Network	No. of nodes	No. of edges	Mean degree <*k*>	Power-law exponent of degree distribution	Mean clustering coefficient *C*
Nor	1436	1215	1.69	−2.75	0.0458
Ade	1801	2281	2.53	−2.23	0.0904
IBD	2478	3457	2.79	−2.22	0.0922
CRC	2318	4988	4.30	−1.85	0.1266

* For Pearson *p*-value *p*_0_ = 0.01. For disease networks numbers given are averaged over 100 8-sample networks.

### CRC Network had the Highest Complexity and was Qualitatively different from the IBD Network

**Figure 3:**
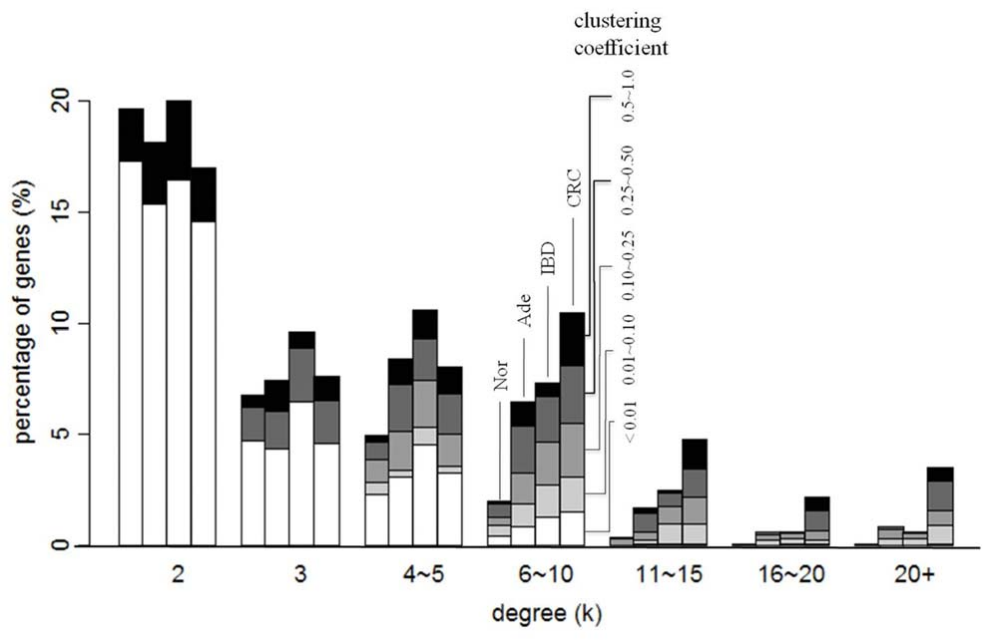
Percentage of genes in a given range of clustering coefficient plotted as a function of degrees in the Nor, Ade, IBD and CRC networks. Genes of degree 1 are not shown. The clustering coefficient of a gene of degree 2 is either 0 or 1. Asterisks indicate *p*-values (by Wilcoxon rank sum tests) relative to Nor: * *p*-value <0.05; ** *p*-value <0.01.

The percentage of hub-like genes increased with disease severity (Figure 3; see Figure S3 for one set of GGINs). For instance, less than 0.5% of the genes in Nor, but more than 10% in CRC, had degrees higher than 11; only CRC had a significant number of genes with degrees 16 or higher; only CRC had a non-negligible percentage of genes with degrees greater than 16 while possessing the highest level of clustering coefficient. Although much larger, the complexity of the IBD network was similar to that of Ade. IBD had more genes of degrees up to 5 than CRC, but fewer high degree nodes and far fewer nodes with high degrees and large clustering coefficients (Figure 3).

### Sizes of Gene Sets of Functional Modules in FFNs Generally Increased with Disease Severity

**Figure 4:**
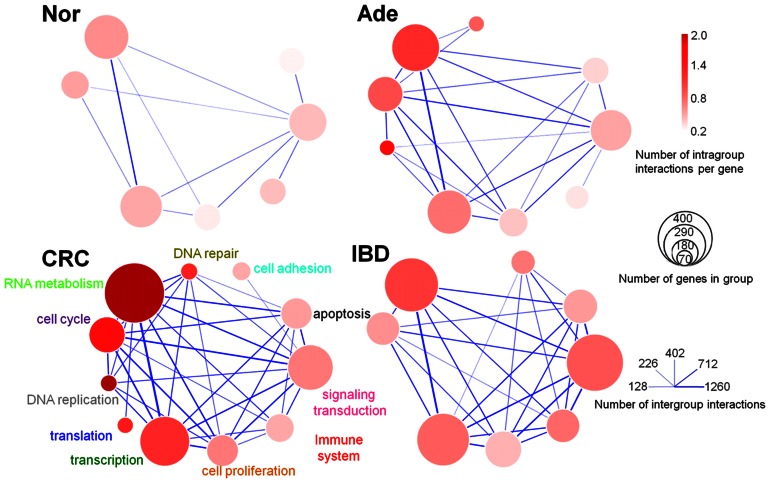
Function-function networks. Nodes are functional modules named after Gene Ontology terms. Functional modules containing less than 70 genes are not shown. The diameter of a module scales with the logarithm of the number of genes in the module. The color shade of a module indicates the number of intra-module gene-gene interactions per gene. The thickness of the edge indicates the number of inter-module gene-gene interactions.

FFNs were reduced from GGINs through DEGs partition according to GO terms (Figure 4; see Table S2 for GO enrichment analysis for the functional modules). Sizes of functional modules in FFNs generally increased with disease severity (Figure S4). The relations Nor<CRC and Ade<CRC held for all 11 functions (the “<” symbol refers to the sizes in gene numbers of functional modules, with p-value less than 10^−4^). The relation Nor<Ade<CRC held in 10 of the 11 functions (the immune system function was the exception), with the trend being especially strong for RNA metabolism, transcription, DNA repair, DNA replication, and cell cycle. In comparison, the relation Nor<IBD held in only six functions: translation, cell adhesion, cell proliferation, immune system, signal transduction and apoptosis. The relation Nor<Ade<IBD did not hold with good statistical support in any of the functions.

### Ade-CRC Pair had Significantly Larger Inter-FFN Percentage Intersections of Functional Link Sets

**Figure 5:**
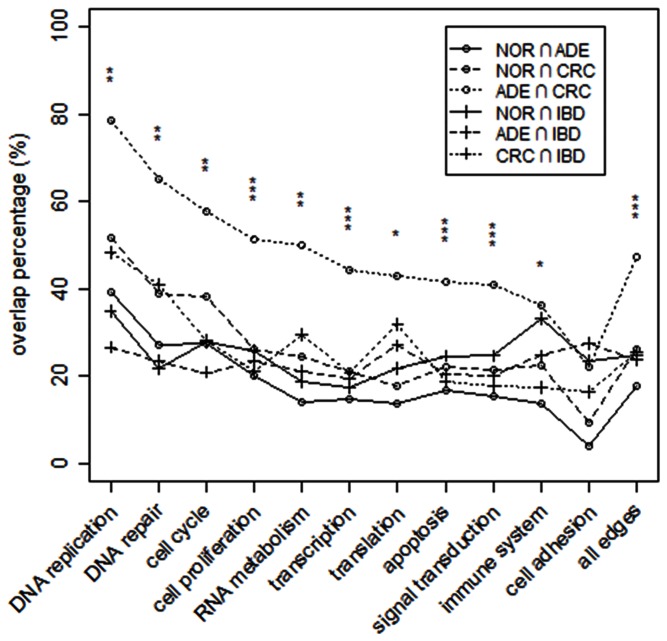
Percentage overlaps of functional modules. 0 For a given functional module, the percentage overlap is expressed as the ration of the number of links (belonging to that module) common to the two networks to the number of links in the smaller partner. Asterisks indicate *p*-values from one-sample Student’s *t*-test of the Ade-CRC intersection versus the other five intersections: for *, **, and ***, *p*-value<10^−2^, 10^−3^, and 10^−4^, respectively.

For every function in a FFN a list of in-function links, namely interactions between two genes in the functional module, was constructed, and percentage Inter-FFN intersections of link sets were computed (Figure 5). The Ade-CRC intersection stood out as an outlier relative to the other five intersections. For almost all functional modules the five intersections were closely bunched at values typically half the size of the corresponding Ade-CRC intersections. Relative to the other five intersections the Ade-CRC intersections had *p*-values of <10^−2^ in all but one of the functions (cell adhesion), and <10^−3^ in seven functions (Figure 5). A similar treatment of the Ade-IBD intersections found that all functions had *p*-values close to unity. The relatively large overlap between DEG sets from Ade and CRC has been noted before [46]–[48].

### Examples of ToP Genes

**Figure 6:**
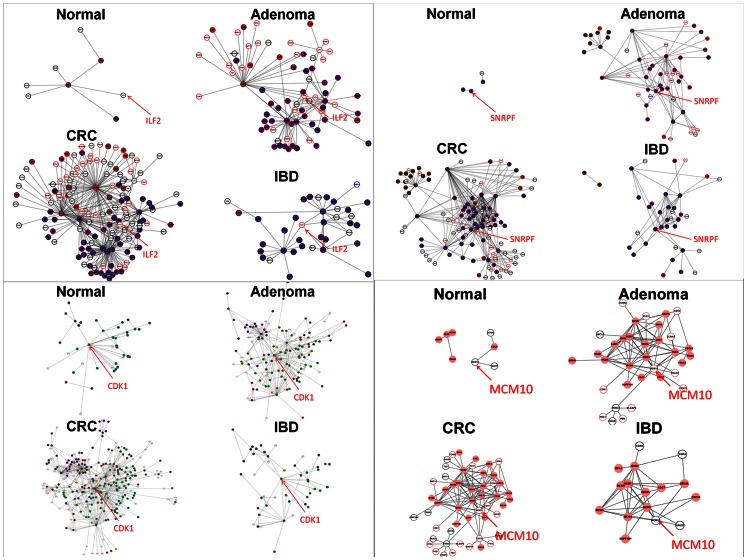
Examples of changes in partial gene networks connected to cancer genes. Partial networks to which the four ToP genes ILF2 (top left), CDK1 (bottom left), SNRPF (top right), and MCM10 (bottom right) separately belong in the Nor, Ade, IBD and CRC networks. In each case, the size of the module connected to the ToP gene increases along the state sequence Nor-Ade-CRC or Nor-IBD-CRC, or both. Nodal trim color code: over-expression, red; under-expression, blue; neutral, black. Nodal color code for GO functions: cell cycle, green; RNA splicing, purple; DNA repair, brown; chromatin remodelling and histone modification, yellow.

A ToP gene was required to have its network connectivity and complexity grew noticeably along a state sequence. Four examples of such genes that code transcription factors (TFs) were the three genes ILF2, CDK1, and SNRPF, curated from both the Ade- and IBD-sequences, and MCM10, exclusively from the IBD-sequence (Figure 6). In each case the predicted gene was a low-degree node in the relatively small Nor network, became a moderate hub in a noticeably grown Ade or IBD network (or both, as the case may be), and finally a super-hub in the large and complex CRC network.

### Discovery of Cancer Genes using the ToP Procedure

The ToP procedure was applied to the Nor-Ade-CRC (or simply Ade) and Nor-IBD-CRC (or IBD) sequences to select cancer genes, yielding lists of 389 and 381 genes, respectively, with 373 genes appearing in both lists (Table S3, Figure S5A). The TPS procedure yielded 134 and 74 genes from the Ade and IBD sequences, respectively, with 67 common to both lists (Table S4, Figure S5B). In comparison, the ToP selected only 7 and 4 genes, respectively, from the CRC-Ade-Nor and CRC-IBD-Nor sequences, and TPS reduced the sets to null sets (data not shown), confirming the two sequences did not exhibit any trend toward a disease state. Application of eBayes and SAM with thresholds *p*-value<0.05 and absolute fold-change>1.5 yielded DEG lists of 2648 and 2666 genes, respectively. Whereas each of the steps in the ToP procedure had an important impact on reducing the pool of candidate genes, the ToP gene requirement was the major limiting factor. For the Ade sequence the requirement that genes encode proteins listed HPRD reduced the number of candidates from 18,267 to 9,122; that it belonged to one of the relevant GGINs, to 3,556; that it was a ToP gene, to 389; that it was a DEG by SAM, to 134. For the IBD sequence the first two reductions were the same, and the corresponding last three numbers were 3,074, 381, and 74 (Figure S6).

### Permutation Tests

**Figure 7:**
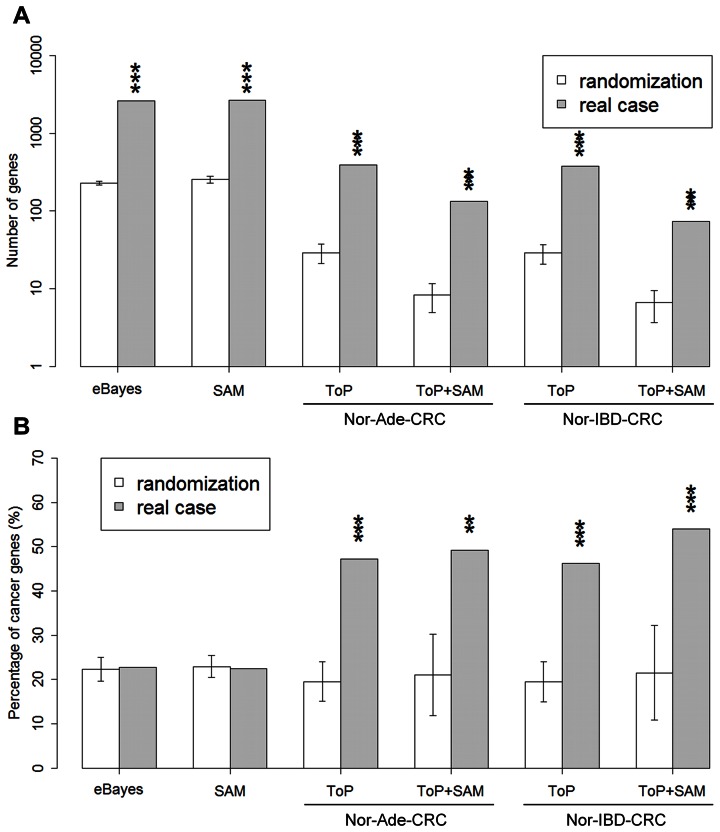
Results from 1000 randomization tests (white box) and in actual cases (gray box). Randomization tests are type-1 for eBayes and SAM, and type-2 for ToP and ToP+SAM (see Methods). (A) Number of genes selected. (B) Percentage of genes listed in *CancerGenes* [40] database among those selected in (A). ***, *p*-value <0.001 for permutation test by randomization; **, *p*-value <0.01; *, *p*-value <0.05.

The *p*-values for permutation tests by randomization of the all the selected genes lists were <0.001 (Figure 7A). The numbers (standard deviation in brackets) of eBayes and SAM DEGs in 1000 type-1 randomizations (see Methods) were 228.81 (13.93) and 255.31 (25.57), respectively (Figure S7A–B). Because randomization destroyed intensity correlation among genes, the 1000 randomizations yielded only 0.42(1.2) genes (Figure S7C), making network construction impossible. For the ToP procedure gene-intensity associated was subject to type-1 randomization and gene-link associated, to type-2 (see Methods). In 1000 randomizations the numbers of genes selected by the ToP and TPS for the Ade sequence were 29.09 (standard deviation 8.18) and 8.31 (3.36), respectively (Figure S8A–B); corresponding number for the IBD sequence were 28.01 (8.15) and 6.58 (2.91) (Figure S8C–D).

### Hit Rates for known Cancer Genes

**Figure 8:**
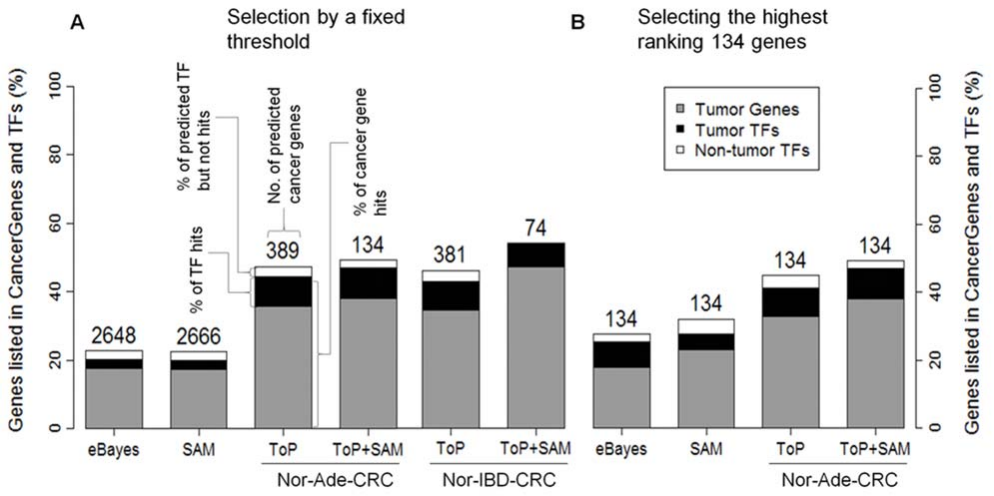
Percentages of selected genes listed in*CancerGenes* [**40**] and gene coding transcription factors (TFs). Non-tumor TF means not listed in *CancerGenes*. (A) In gene set selected by statistical threshold. (B) In top 134 genes in gene sets. Numbers given above bars indicate total number genes in set.

Distribution of hit rates for known cancer related genes in gene selected in 1000 randomization of conventional methods (eBayes and SAM; Figure S7D–E) and ToP based methods (Ade-ToP, Ade-TPS, IBD-ToP, and IBD-TPS; Figure S8E–H) all have averages in the 19%–23% range, an expected value in view of the 3,165 cancer related genes among the 18,267 genes on a HG-U133 Plus 2.0 array. The hit rates of the real cases (permutation test *p*-value by randomization in brackets) were 23% (0.422), 22% (0.547), 47% (<0.001), 50% (0.008), 51% (0.008), and 54% (<0.001), respectively (Figure 7B). In comparison, the average hit rate of selected genes in all randomization tests was ∼20% (Figure S8). The hit rates for the top 134 genes from eBayes and SAM were 27% and 33%, respectively (Figure 8). The combined Ade and IBD TPS list had 141 predicted cancer genes, of which 67 came exclusively from Ade, 67 were common to Ade and IBD, and 7 came exclusively from IBD (Table S3). GO enrichment analysis showed that the GO terms nuclear lumen, cell cycle and nucleoside binding were the most enriched, involving 51%, 33% and 34%, respectively, of the genes (Table 2). Sixty-seven of the 141 genes were known cancer genes, of which 27, 39, and 1, respectively, came from Ade only, were common to Ade and IBD, and came from IBD only (Table S4).

**Table 2:** Gene ontology enrichment analysis for predicted cancer genes.

Gene Ontology	Class	Genes (%)	*p*-value	Adjusted *p*-value (BH)
Nuclear lumen	CC	71 (51%)	2.60e-33	6.40e-31
Cell cycle	BP	46 (33%)	1.40e-23	9.50e-21
Nucleoside binding	MF	47(34%)	1.50e-12	2.50e-10

### CRC Cancer Genes and Transcription Factors

Forty-eight of the 141 genes had been reported to be CRC cancer genes, of which 15, 32, and 1, respectively, came from Ade only, were common to Ade and IBD, and from IBD only (Table 3). The percentage of transcription factor (TF)-encoding genes among the selected genes varied depending on method used (Figure 8A). In the case of the top 134 genes, the number of TF genes ranged from 10 to 17 (Figure 8B). Among the 141 TPS genes, 16 were transcription factor (TF)-encoding (Table 4), of which 12 were listed in *CancerGenes* [40] and 11, including the 3 not listed in *CancerGenes*, had been cited in the literature as CRC associated (Table 3). PML, listed in *CancerGenes* and cited in the literature as CRC related, was the only TF among the 16 TFs that came exclusively from the IBD sequence; the four TFs CEBPB, E2F5, MYC, and RUVBL1 were common to both the Ade and IBD sequences; the remaining 11 came exclusively from the Ade sequence (Table 4).

**Table 3:** The 48 genes among the 141 predicted cancer genes known in the literature as diagnostic or prognostic markers for CRC, or are reported to be associated with them (Table S5).

Gene symbol	Gene name	No. of researchpapers	Diagnosticmarkers	Prognostic markers
Sustaining proliferative signaling				
AURKB	aurora kinase B	3		V
BUB1B	budding uninhibited by benzimidazoles 1 homolog beta (yeast)	4		V
CCND1	cyclin D1	3		
CDC25A	cell division cycle 25 homolog A (S. pombe)	4		V
CDC25B	cell division cycle 25 homolog B (S. pombe)	2		V
CDK1	cyclin-dependent kinase 1	4		V
CDK2	cyclin-dependent kinase 2	>20		
CDK4	cyclin-dependent kinase 4	6		V
CDK8	cyclin-dependent kinase 8	3		V
CENPA	centromere protein A	3		
E2F5*	E2F transcription factor 5, p130-binding	4		
HMGA1	high mobility group AT-hook 1	2		
MAD2L1	MAD2 mitotic arrest deficient-like 1 (yeast)	2		V
MKI67	antigen identified by monoclonal antibody Ki-67	4		V
MYC*	v-myc myelocytomatosis viral cancer gene homolog (avian)	5	V	
PML	promyelocytic leukemia	3	V	
PLK1	polo-like kinase 1 (Drosophila)	3		V
PTPN11	protein tyrosine phosphatase, non-receptor type 11	3		
SKP2	S-phase kinase-associated protein 2 (p45)	2		V
TUBB	tubulin, beta	5		
Resisting cell death				
BCL2*	B-cell CLL/lymphoma 2	7		V
BIRC5	baculoviral IAP repeat-containing 5	>20		V
KAT2B	K(lysine) acetyltransferase 2B	1		
HSPH1	heat shock 105 kDa/110 kDa protein 1	3	V	
RFC2*	replication factor C (activator 1) 2, 40 kDa	1		
TRAP1	TNF receptor-associated protein 1	3		
Inducing agiogenesis				
PECAM1	platelet/endothelial cell adhesion molecule	2		
MMP2	matrix metallopeptidase 2 (gelatinase A, 72 kDa gelatinase,72 kDa type IV collagenase)	7		V
MMP9	matrix metallopeptidase 9 (gelatinase B, 92 kDa gelatinase,92 kDa type IV collagenase)	16		V
Activating invasion and metastasis				
CEBPB	CCAAT/enhancer binding protein (C/EBP), beta	3		
CSE1L	CSE1 chromosome segregation 1-like (yeast)	5	V	
PLAU	plasminogen activator, urokinase	4		V
PSAT1	phosphoserine aminotransferase 1	1		
SNRPF	small nuclear ribonucleoprotein polypeptide F	3		
SPARC	secreted protein, acidic, cysteine-rich (osteonectin)	7		V
TGFB1*	transforming growth factor, beta 1	11	V	
Enabling replicative immortality				
PARP1	poly (ADP-ribose) polymerase 1	1	V	
TOP2A	topoisomerase (DNA) II alpha 170 kDa	2		
Epigenetic switching				
EZH2	enhancer of zeste homolog 2 (Drosophila)	4		V
HDAC2	histone deacetylase 2	9	V	
NAT10	N-acetyltransferase 10 (GCN5-related)	13		
PRMT1	protein arginine methyltransferase 1	2		V
Hyperactivation of fatty acid synthase				
FASN	fatty acid synthase	3		V
Genome Instability and Mutation				
ATR	ataxia telangiectasia and Rad3 related	3		
MRE11A	MRE11 meiotic recombination 11 homolog A (S. cerevisiae)	4		
MSH2	mutS homolog 2, colon cancer, nonpolyposis type 1 (E. coli)	>20		
Deregulating cellular energetics				
PKM2	pyruvate kinase, muscle	4	V	
Avoiding immune destruction				
ILF2	interleukin enhancer binding factor 2, 45 kDa	2		

**Table 4:** The sixteen transcription factors predicted to be cancer genes in this study.

TF	Degree			Clustering coefficient			Student’s *t*-test (Ade/IBD v.s. Nor)		Student’s *t*-test (CRC v.s. Nor)			OMIM	Listed in *CancerGenes* [40]
	Nor	Ade/IBD	CRC	Nor	Ade/IBD	CRC	*p*-value	fold change	*p*-value	fold change			
Ade only													
^+^CDK2	1	22	48	0	0.05	0.05	5.58e-03	1.51	6.88e-02	1.33		116953	YES
^+^EZH2	0	6	11	0	0.07	0.02	1.63e-02	1.71	7.74e-02	1.46		601573	YES
^+^HDAC2	0	3	35	0	0	0.07	1.47e-05	1.53	3.54e-02	1.33		605164	YES
^+^HMGA1	0	0	6	0	0	0.13	<1e-06	1.78	9.47e-02	1.25		600701	YES
^+^KAT2B	2	3	8	0	0	0	1.35e-03	0.55	1.60e-01	0.82		602303	YES
SUPT16H	1	5	8	0	0.4	0.32	5.39e-05	1.58	6.46e-02	1.21		605012	YES
TRIM28	0	4	8	0	0.17	0.11	1.47e-05	1.56	4.79e-02	1.33		601742	YES
YEATS4	0	3	12	0	0.67	0.68	1.02e-03	1.68	5.50e-02	1.48		602116	YES
^+^CDK1	21	39	68	0.01	0.03	0.01	3.25e-03	2.27	5.34e-02	1.92		116940	NO
^+^ILF2	1	7	12	0	0.29	0.64	<1e-06	1.83	1.58e-02	1.49		603181	NO
^+^SNRPF	1	10	20	0	0.27	0.38	4.28e-04	1.52	6.97e-02	1.37		603541	NO
Ade & IBD													
^+^CEBPB	1	1/6	7	0	0/0	0.05	3.24e-04/7.92e-05	1.80/1.20	5.15e-03	2.48		189965	YES
^+^E2F5	1	1/0	7	0	0/0	0.29	<1e-06/1.76e-03	1.99/0.55	5.56e-03	1.89		600967	YES
^+^MYC	1	0/1	21	0	0/0	0.03	<1e-06/3.1e-01	3.05/0.13	2.26e-02	2.14		190080	YES
RUVBL1	0	2/0	17	0	1/0	0.26	<1e-06/2.8e-03	2.10/0.41	8.37e-03	1.78		603449	YES
IBD only													
^+^PML	0	0	11	0	0	0.05	<1e-06	0.85	2.00e-02	0.42	102578		YES

In each case, the degree of the TF increases in the progression Nor to Ade to CRC. TF’s in the first column marked by “+” have been reported in the literature as being associated with CRC and appear in Table 3.

### Biomarkers for Early Diagnostic Detection of CRC

Among the 141 predicted TPS cancer genes 13 were identified as markers for early diagnosis of CRC; 11 for detection in the Ade state, of which 9 came exclusively from the Ade sequence and 2 were common to both sequences, and 2, for detection in the IBD state and also common to both sequences (Table 5). In each case the candidate either did not appear or appeared as a single-link gene in (the) Nor (network), but blossomed into one having five or more links and were strongly expressed (*p*-value <0.0001) in Ade or IBD, as the case may be, and proceeded to become a substantial hub in CRC.

**Table 5:** Predicted candidate diagnostic markers for early detection of CRC in the Ade or IBD state.

Gene	Degree				Clustering coefficient			Student’s *t*-test (Ade/IBD vs. Nor)		Student’s *t*-test(CRC vs. Nor)		ANOVA*p*-value	*Cancer Genes* [40]
	Nor		Ade	CRC	Nor	Ade	CRC	*p*-value	fold change	*p*-value	fold change		
Ade													
*SUPT16H	1		5	8	0	0.4	0.32	5.39e-05	1.58	6.46e-02	1.21	1.00e-05	YES
^#^PRMT5	0		6	8	0	0.33	0.25	<1e-06	1.78	2.77e-02	1.34	5.61e-06	YES
NOLC1	1		9	28	0	0.78	0.57	2.85e-05	1.75	2.30e-02	1.44	4.22e-05	NO
^#$^PSAT1	0		5	18	0	0.4	0.67	<1e-06	6.15	2.70e-03	5.62	3.54e-05	NO
CCT7	1		9	18	0	0.56	0.41	8.43e-05	1.54	2.58e-02	1.4	1.05e-03	NO
CCT4	0		7	17	0	0.71	0.46	<1e-06	1.57	1.62e-02	1.45	1.25e-04	NO
*^#^ILF2	1		7	12	0	0.29	0.64	<1e-06	1.83	1.58e-02	1.49	2.13e-05	NO
^$^CCT3	0		5	11	0	0.6	0.67	<1e-06	1.84	1.45e-02	1.55	1.81e-04	NO
DARS	0		6	10	0	0.33	0.47	4.89e-05	1.62	3.71e-02	1.43	1.37e-03	NO
CCT8	1		7	9	0	0.76	1	1.91e-05	1.51	1.43e-02	1.42	4.56e-04	NO
GEMIN6	0		5	9	0	0.5	0.72	<1e-06	1.56	1.91e-02	1.39	5.28e-04	NO
IBD													
^$#^*CEBPB		1	6	7	0	0.00	0.05	7.92e-05	1.20	5.15e-03	1.31	1.01E-03	YES
^$#^PLAU		0	5	6	0	0.00	0.07	3.22e-05	1.75	7.44e-03	1.43	7.76E-05	YES

A biomarker for early detection in the Ade state is not a DEG in the IBD state, and vice versa. The hash and asterisk superscripts indicate the gene also appears in Tables 3 and 4, respectively. Genes with a $ superscript are common to the ToP+SAM lists for the Ade and IBD sequences; genes without are from the Ade sequence only.

## Discussion

Most noticeable about the GGINs was that their sizes and complexities grew with the severity of disease (Figure 2) in ascending order: Nor, Ade, IBD, and CRC. The IBD network had slightly more genes but far fewer links than CRC. In the three metrics that measured network complexity, IBD closely resembled Ade, placing the two midway between Nor and CRC (Table 1). From this we infer that normal and healthy cells operate under optimal and the most efficient conditions, whereas systemically diseased cells such as cancer cells are the extreme opposite.

The ToP procedure succeeded in confirming both Ade and IBD sequences as state sequences trending to cancer, while showing the sequences CRC-Ade-Nor and CRC-IBD-Nor did not. In comparison, the much simpler method of examining overlaps of functional modules (Figure 5) alone was not a reliable identifier of ToP sequence: it suggested Ade sequence as ToP, but not the IBD sequence. The procedure also identified candidate cancer genes with high efficiency. However, the IBD sequence had a significantly weaker trend toward CRC than Ade. Although the IBD network was much larger than the Ade network (Table 1), exclusive IBD-sequence contribution to various categories of cancer genes was much smaller than that from the Ade sequence (Table 6). This seems to suggest that unlike Ade, which is essentially a way station to CRC, IBD may or may not lead to CRC. As an indication of this weaker trend, the permutation-test *p*-value for the 74 IBD-TPS genes in 1000 topology-conserving type-3 randomizations (weaker than a true randomization; see Methods) was close to unity. In comparison, the p-value for the 134 Ade-TPS genes in a similar test was <0.001.

**Table 6:** Types of cancer genes contributed by the Ade and IBD state-sequences.

From TPS list generated by	Predicted cancer genes	In *Cancergenes* [40]	Reported in literature as CRC-related	TFs	Markers for early detection (state of detection)
Ade sequence	67	27	15	11	9 (Ade)
Both Ade & IBD sequences	67	39	32	4	2 (Ade) 2 (IBD)
IBD sequence	7	1	1	1	0
Total	141	67	48	16	13

Although there are unknown errors in array data, it is generally acknowledged that the associated noise is much smaller than variations in data due to heterogeneity in patients. Assumption we used to construct the GGINs and to identify ToP genes may have its own sources of errors. For instance, GGIN construction might be improved by incorporating sub-cellular localization data [49], and selection rules for ToP genes could be further refined. On the other hand, the fact that our ToP gene lists had permutation test *p*-values less than 0.001 and had high hit rates for known cancer genes provides assurance that most of the selected genes were not chance results.

A surprise of this study was that although eBayes and SAM were just as statistically robust as ToP in identifying differentially behaving genes as potential biomarkers, the two standard methods did not select cancer genes with statistical significance (*p*-value ∼0.5), a task ToP did very well (*p*-values <0.001) (Figure 7). The inference is that a biomarker, even in cancer related diseases, need not be cancer causing; it may be simply a symptom of cancer. The better performance by ToP in identifying cancer genes confirmed our supposition that motivated the design of the ToP procedure: cancer genes tended to be hubs in GGINs.

A majority of the 48 predicted genes already known to be CRC associated (Table 3) were on common CRC pathways: proliferating signals, resisting cell death, inducing angiogenesis, invasion and metastasis. Four genes had functions in epigenetic switching: histone deacetylase 2 (HDAC2), enhancer of zeste homolog 2 (EZH2), N-acetyltransferase 10 (NAT10), protein arginine methyltransferase 1(PRMT1). Five, the transforming growth factor beta 1 (TGFB1), B-cell CLL/lymphoma 2 (BCL2), replication factor C (activator 1) 2, 40kDa (RFC2); E2F transcription factor 5, p130-binding (E2F5), and v-myc myelocytomatosis viral cancer gene homolog (MYC) were among the cancer “hallmark” genes discussed in [50]. TGF-beta is best known for its anti- proliferative and apoptosis inducing effects. In many late-stage tumors, TGF-beta signalling is redirected away from suppressing cell proliferation to activating a cellular EMT (the epithelial-to-mesenchymal transition) process, and confers on cancer cells traits associated with angiogenesis and migration [51]–[53]. In the event, the real anti-apoptotic signals are conducted by the Bcl-2 family of regulatory proteins including Bcl-xL, Bcl-w and A1, acting largely by binding to suppress two pro-apoptopic proteins (Bax and Bak) [54]. MYC and E2F are up-regulated in mitogenic tumors in order to sustain proliferative signals. These genes have diverse effects on tumor progression but share two general principles, higher levels in a specific signal and versatile functions across different cancer signals, and illustrate the principle that distinct cancer hallmarks can be co-regulated by the same transforming agent. For example, TGF-beta from tumor cells not only contributes to invasion and migration, but also plays a role in evading immune destruction [55]. MYC induces angiogenesis directly and drives proliferative signaling [56]. PML is the only CRC associated gene curated exclusively from the IBD sequences. It is often involved in the translocation with the retinoic acid receptor alpha gene associated with acute promyelocytic leukemia. The gene encodes a phosphoprotein that localizes to nuclear bodies and has many functions, including as a transcription factor and tumor suppressor [57]. Eight of the predicted 48 CRC cancer genes have been studied as diagnostic markers and 20, prognostic markers (Table 3).

The vast majority of the 141 predicted cancer genes were up-regulated. Only 12 were down-regulated, of which 6 were from the 7 exclusive IBD-sequence genes. Four of the down-regulated genes were known cancer genes: KAT2B (from the Ade sequence only), BCL2, IQGAP2 (from Ade & IBD), and PMT (from IBD only). KAT2B encodes K (lysine) acetyltransferase 2B, also known as P300/CBP-associated factor, a protein that suppresses the adenoviral oncoprotein E1A by counteracting its mitogenic activity [58]. BCL2 encodes a family of proteins that regulate and contribute to apoptosis; some members of the family are anti-apoptosis while others are pro-apoptosis [59], [60]. IQGAP2 encodes a member of the IQGAP (IQ motif containing GTPase activating protein) family. It interacts with other biomolecules to regulate cell morphology and motility [61]. PMT encodes promyelocytic leukemia, a phosphoprotein that localizes to nuclear bodies where it functions as a transcription factor and tumor suppressor [57].

Three of the 16 TF-encoding genes in Table 4, cyclin-dependent kinase 1 (CDK1), small nuclear ribosomal polypeptide F (SNRPF), and interleukin enhancer binding factor 2 (ILF2), were not listed in the *CancerGenes* [40]. However, they show strong ToP characteristics (Figure 6) and have been reported in the literature as being CRC related (Table S5). We therefore view them as novel cancer genes for CRC. An analysis of the GO enrichment in the CRC network of protein modules regulated by the 16 TFs (Figure S9) indicated that cell cycle (with 7 and 8 TFs showing strong and moderate over-representation, respectively), DNA repair (4 and 11), RNA splicing (6 and 5), chromatin remodeling (null and 13), histone modification (null and 13), DNA methylation (null and 5), angiogenesis (null and 1), and inflammatory response (null and 1) show over-representation. These may: reflect the instability of the genomes of tumorous cells that facilitates the selection of cells for their abilities to proliferate and invade and to evade host immune systems (cell cycle and DNA repair) [62]; reflect the preponderance of alteration in epigenetic regulation of gene expression, a frequent event in human cancer (the three epigenetic functions) [63]; suggest that tumorous cells utilize alternative splicing of mRNA transcripts to generate abnormal genomic complexity thereby hampering the effectiveness of tumor suppressor genes including APC, TP53, and BRCA1 [64] or cause erroneous RNA splicing in cancer cells (RNA splicing) [65]. Modules regulated by the three novel TFs are highly or moderately overrepresented in RNA splicing, DNA repair, RNA splicing. In addition, those by SNRPF are moderately overrepresented in chromatin remodelling.

Because ToP traces the network properties of genes through sequences of states, starting from the healthy Nor through an intermediate state (Ade and IBD in the present case) to the final disease state (CRC), it naturally lends itself as a tool for screening genes at the intermediate state for early detection of the development toward the eventual disease state. We identified 13 such genes, 11 markers in Ade and 2 in IBD (Table 5), 5 of which, PRMT5, PSAT1, ILF2, CEBPB, and PLAU were known to be CRC related (Table 3). Among the predicted early markers in Ade, SUPT16H (FACT complex subunit SPT16) is a histone interacting protein that facilitates chromatin transcription; it is a TF and is listed in *CancerGenes* (Table 4). PRMT5 (Histone-arginine N-methyltransferase 5) has been reported to be CRC associated (Table 3), is not listed in *CancerGenes* but its homolog PRMT1, which is (Table 4). Both belong to the PRMT family of genes involved in post-translational arginine methylation, and both are believed to regulate the transcriptional elongation properties of SUPT5H, a homolog of SUPT16H. The emergence of SUPT16H and PRMT5 as early markers may indicate tumorigenic epigenetic modification begins at an early stage. PSAT1, which encodes a phosphoserine aminotransferase, is reported to be CRC associated (Table 3); its overexpression stimulates cell growth and increases chemoresistance in CRC cells [62]. The novel TF-encoding cancer gene ILF2 is the only early marker that appears in both Tables 4 and 5. Its low activity in Nor and drastically increased activity in Ade has already been noted (Figure 6). A slightly less stringent selection criterion – increasing the *p*-value threshold from 0.0001 to 0.0005– would qualify a second novel TF-encoding cancer gene, SNRPF (Figure 6 and Table 4), as an early detection marker. Four members of the CCT gene family that code various subunits of the chaperonin containing T-complex protein, CCT3, CCT4, CCT7, and CCT8, are predicted early markers. None are listed in *CancerGenes* or have been reported to be cancer associated. However, our results showed CCT7 to be the most active, and CCT4 and CCT8 among the most active, early markers in Ade, and CCT7 and CCT4 to be significant hubs in CRC (Table 5). The early marker that develops into the most significant hub was NOLC1. This gene, not known to be associated with CRC, encodes the nucleolar and coiled-body phosphoprotein 1, has been reported to be an enhancer of nasopharyngeal carcinoma progression, and is essential for TP53 to regulate MDM2 expression [65]. CEBPB and PLAU were the only predicted markers for early detection of CRC in the IBD. The PLAU gene encodes plasminogen activator, a serine protease involved in degradation of the extracellular matrix and possibly tumor cell migration and proliferation [66]. The CEBPB gene is an important transcriptional activator that plays a role in the regulation of acute-phase reaction, inflammation and hemopoiesis [67], [68].

### Summary and Conclusion

We summarize the main results in this report: (i) We built GGINs for the four states Nor, Ade, IBD, and CRC. In terms of interaction number and network complexity, Nor was the smallest and least complex, CRC was at the opposite extreme, and Ade and IBD were intermediates. (ii) We devised a ToP procedure based on using changes in state-dependent network complexity of individual genes for identifying genes that exhibited a trend of disease progression, and used the procedure to identify sizable sets of ToP and TPS (i.e., ToP+SAM) genes (permutation test *p*-value <0.001) from the two state sequences Nor-Ade-CRC (the Ade sequence) and Nor-IBD-CRC (the IBD sequence). (iii) About 50% of the ToP (permutation test *p*-value <0.01) and TPS (permutation test *p*-value <0.001) genes were known cancer genes, compared to about 22% of DEGs selected by SAM (permutation test *p*-value ∼0.5). (iv) TPS genes from the Ade sequence numbered 134, those from the IBD sequence numbered 74; the intersection of the two lists had 67 genes. (v) IBD is a weaker precursor to CRC than Ade; of the 13 genes identified as markers for early diagnosis of CRC, 11 was for detection in the Ade state and 2, in the IBD state.

We have shown ToP to be potentially powerful procedure for predicting cancer genes from gene expression data. Our results should be subject to experimental tests. Because every predicted cancer genes also had a predicted growing (or shrinking in a few cases) gene network underlying it, the prediction may be validated (or not) by a suitably timed series of tests. Such tests could provide new insights to colorectal tumorigenesis. Our early detection marker may also similarly be validated, if samples tracing the development of Ade or IBD patients with and without the marker gene up-regulated are made available. We believe the ToP procedure can be usefully applied to other types of cancers and other systems diseases. Ultimately, we envision the ToP approach developed into a routine tool used in the early detection and the diagnostic of cancer (and other systems diseases), and for drug discovery for systems cancer treatment.

## Supporting Information

Figure S1 **ANOVA** ***p*** **-values and fold-changes determined with the SAM algorithm of 84 genes (in 36 colon biopsies) whose significances were verified Real-time PCR data** [**15**] **.**
(TIF)

Figure S2 **Hierarchical clustering for 2,666 differential expressed genes, or DEGs.** The genes are classified according to GO terms. Color bar gives normalized log2-intensities of genes.
(TIF)

Figure S3 **Genes in the giant clusters of the** ***p*** **_0_ = 0.01 networks are color-coded according the Gene Ontology functional modules.**
(TIF)

Figure S4 **Numbers of genes in GO classification in the** ***p*** **_0_ = 0.01 Nor, Ade, IBD and CRC networks.** For Ade, CRC, and IBD, error bars are obtained from bootstrapping 100 times eight out of fifteen chips. Asterisks indicate *p*-values from one-sample Student’s *t*-tests between a disease state and Nor: for *, **, ***, and ****, *p*-value <10^−4^, 10^−8^, 10^−12^, and 10^−16^, respectively.
(TIF)

Figure S5 **Gene sets selected in the ToP and ToP+SAM (TPS in text) procedures from the Nor-Ade-CRC and Nor-IBD-CRC sequences, and their intersections.**
(TIF)

Figure S6 **Size of gene set after each stage of screening in the ToP procedure.**
(TIF)

Figure S7 **Results from 1000 type-1 randomization tests (see Methods) and in actual cases (red lines).** **(**A–C) Distribution of number of selected genes. (D–E) Distributions of percentages of selected genes listed in *CancerGenes* [40].
(TIF)

Figure S8 **Results from 1000 type-2 randomization tests (see Methods) and in actual cases (red lines). (**A–D) Distribution of number of selected genes. (E–H) Distributions of percentages of selected genes listed in *CancerGenes* [40].
(TIF)

Figure S9 **Analysis of gene ontology enrichment in the CRC network of protein modules (right-hand column) regulated by the 16 TFs (bottom) selected by ToP+SAM.**
(TIF)

Table S1 **Gene ontology enrichment results for six DEGs clusters.**
(XLS)

Table S2 **Gene ontology enrichment analysis for the four networks.**
(XLS)

Table S3 **The 397 predicted cancer genes curated by the ToP procedure.** Genes in the first column marked by “*”, exclusively from the Nor-Ade-CRC sequence; Genes marked by “#”, exclusively from the Nor-IBD-CRC sequence; Genes without marks, from both.
(XLS)

Table S4 **The 141 predicted cancer genes curated from the ToP+SAM procedure.** Genes in the first column marked by “*”, exclusively from the Nor-Ade-CRC sequence; Genes marked by “#”, exclusively from the Nor-IBD-CRC sequence; Genes without marks, from both.
(XLS)

Table S5 **References on the 48 CRC-related genes among the 141 predicted cancer genes.** Genes are grouped under GO terms. The symbol #, exclusively from the Nor-Ade-CRC sequence; &, exclusively from the Nor-IBD-CRC sequence; $, from both. PML is the only "&" gene.
(XLS)
