# Supplementary figures and images for: ToP: A Trend-of-Disease-Progression Procedure Works Well for Identifying Cancer Genes from Multi-State Cohort Gene Expression Data for Human Colorectal Cancer
Source: PLoS One. 2013 Jun 14;8(6):e65683. doi: 10.1371/journal.pone.0065683 (PMC3683052; doi:10.1371/journal.pone.0065683)

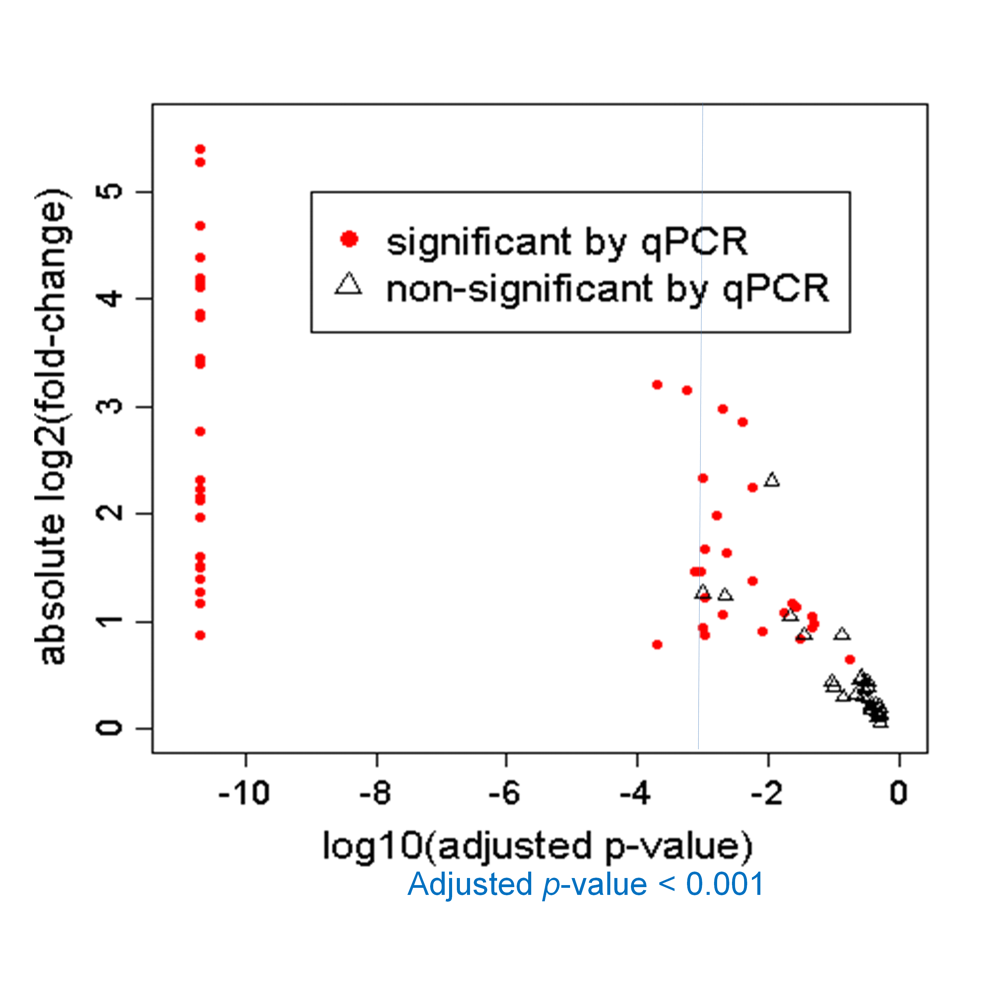

Supplement: Figure S1 — ANOVA p -values and fold-changes determined with the SAM algorithm of 84 genes (in 36 colon biopsies) whose significances were verified Real-time PCR data [15] . (TIF) [file pone.0065683.s001.tif]

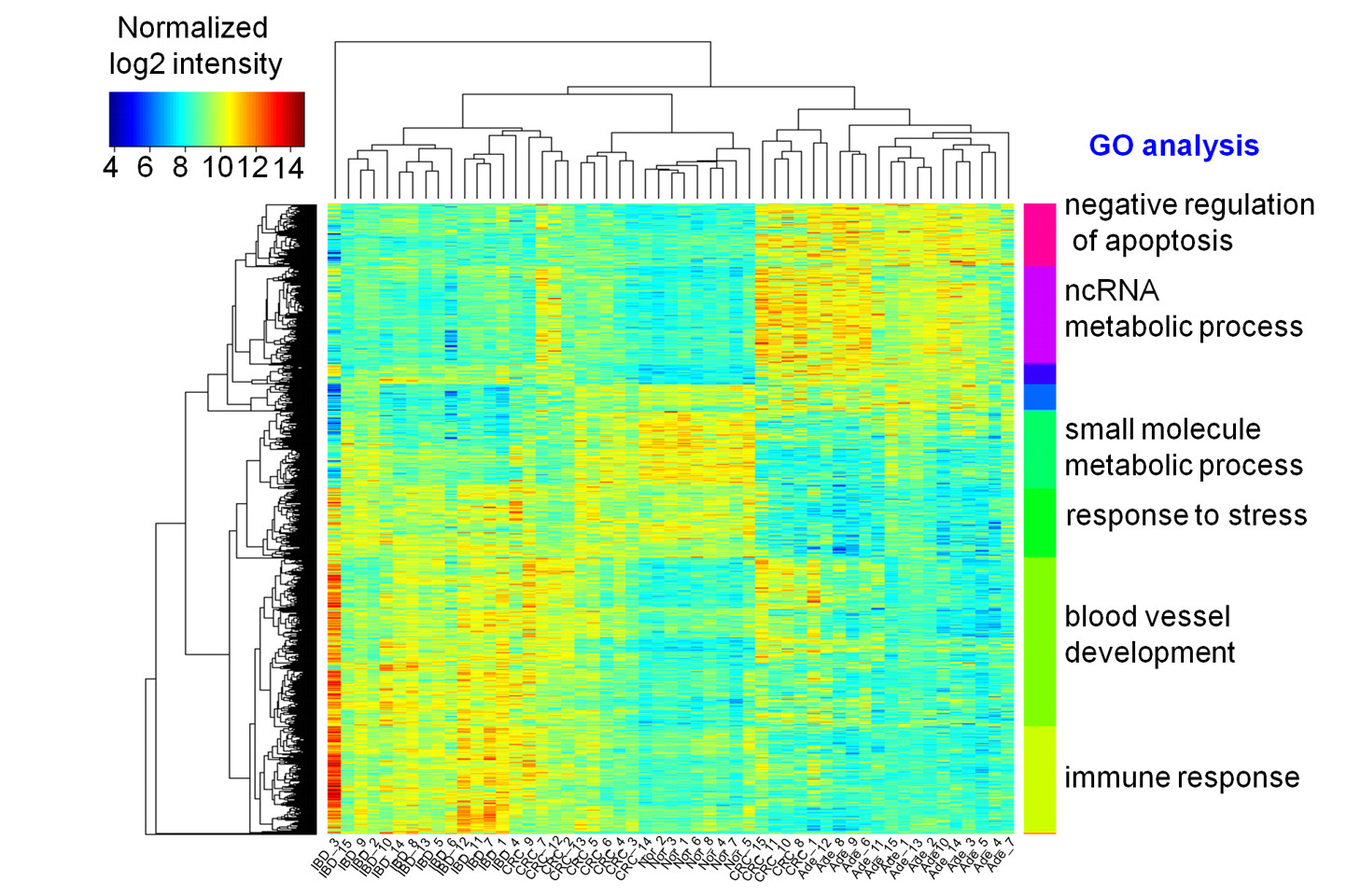

Supplement: Figure S2 — Hierarchical clustering for 2,666 differential expressed genes, or DEGs. The genes are classified according to GO terms. Color bar gives normalized log2-intensities of genes. (TIF) [file pone.0065683.s002.tif]

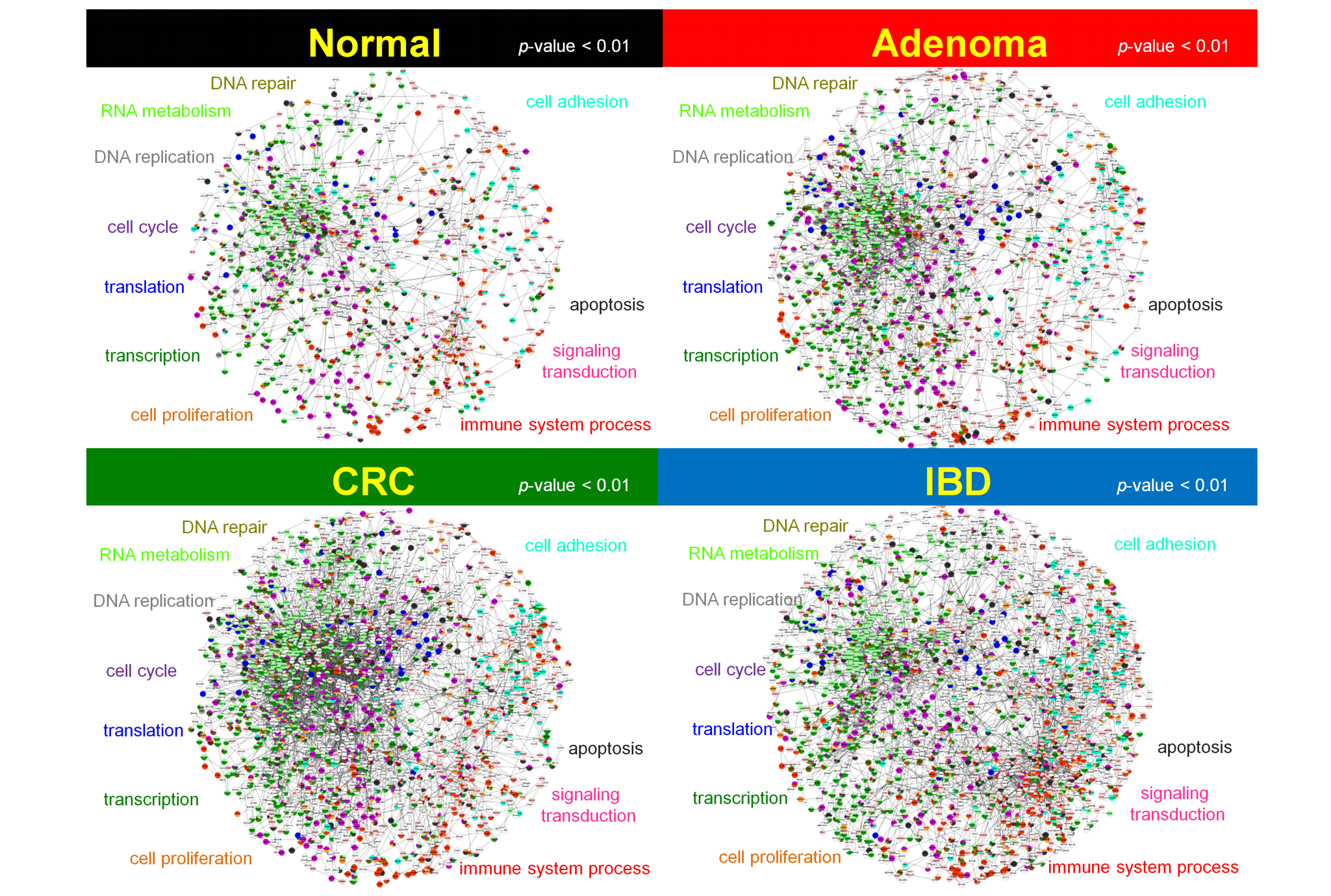

Supplement: Figure S3 — Genes in the giant clusters of the p 0 = 0.01 networks are color-coded according the Gene Ontology functional modules. (TIF) [file pone.0065683.s003.tif]

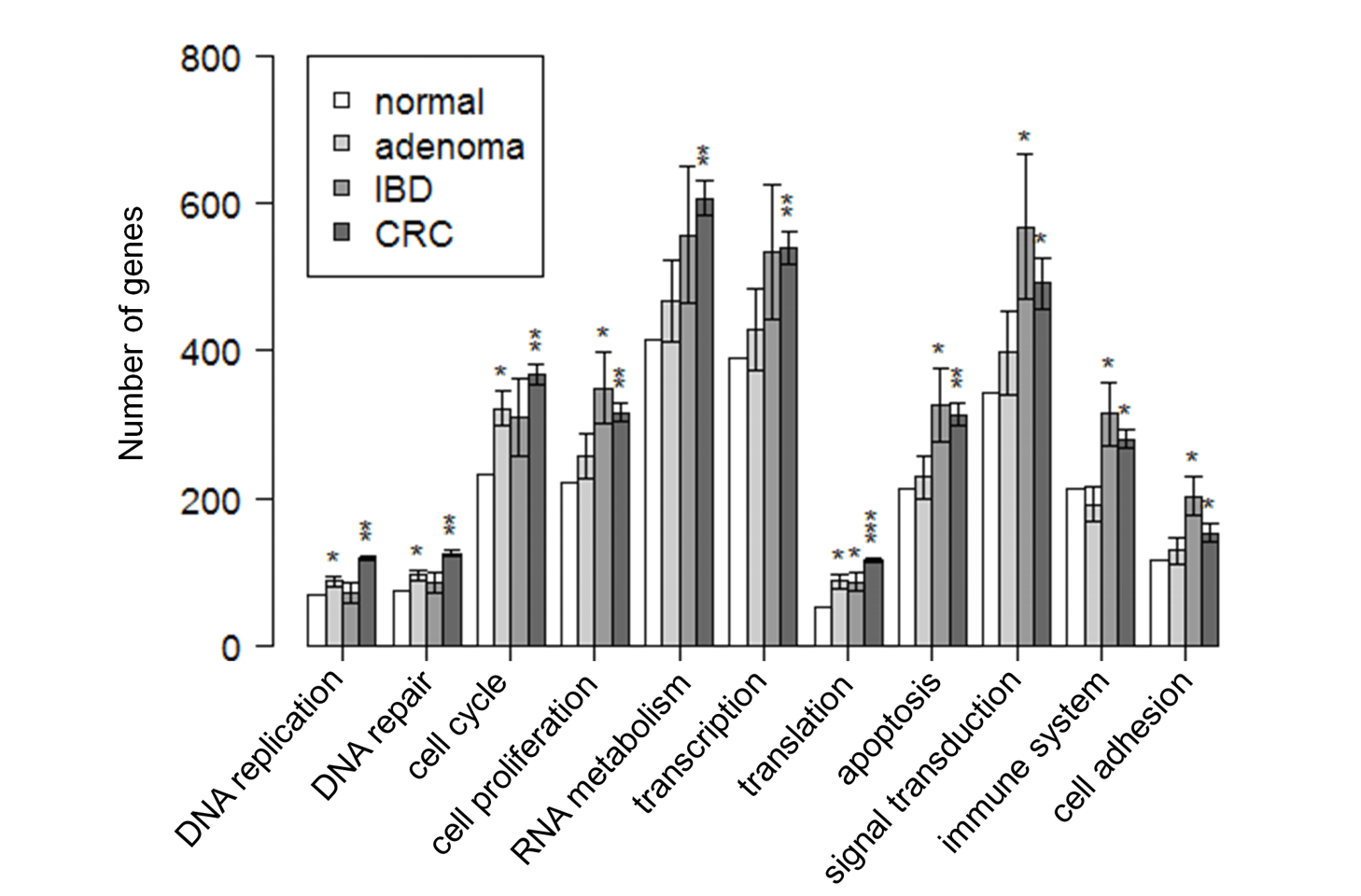

Supplement: Figure S4 — Numbers of genes in GO classification in the p 0 = 0.01 Nor, Ade, IBD and CRC networks. For Ade, CRC, and IBD, error bars are obtained from bootstrapping 100 times eight out of fifteen chips. Asterisks indicate p-values from one-sample Student’s t-tests between a disease state and Nor: for *, **, ***, and ****, p-value <10−4, 10−8, 10−12, and 10−16, respectively. (TIF) [file pone.0065683.s004.tif]

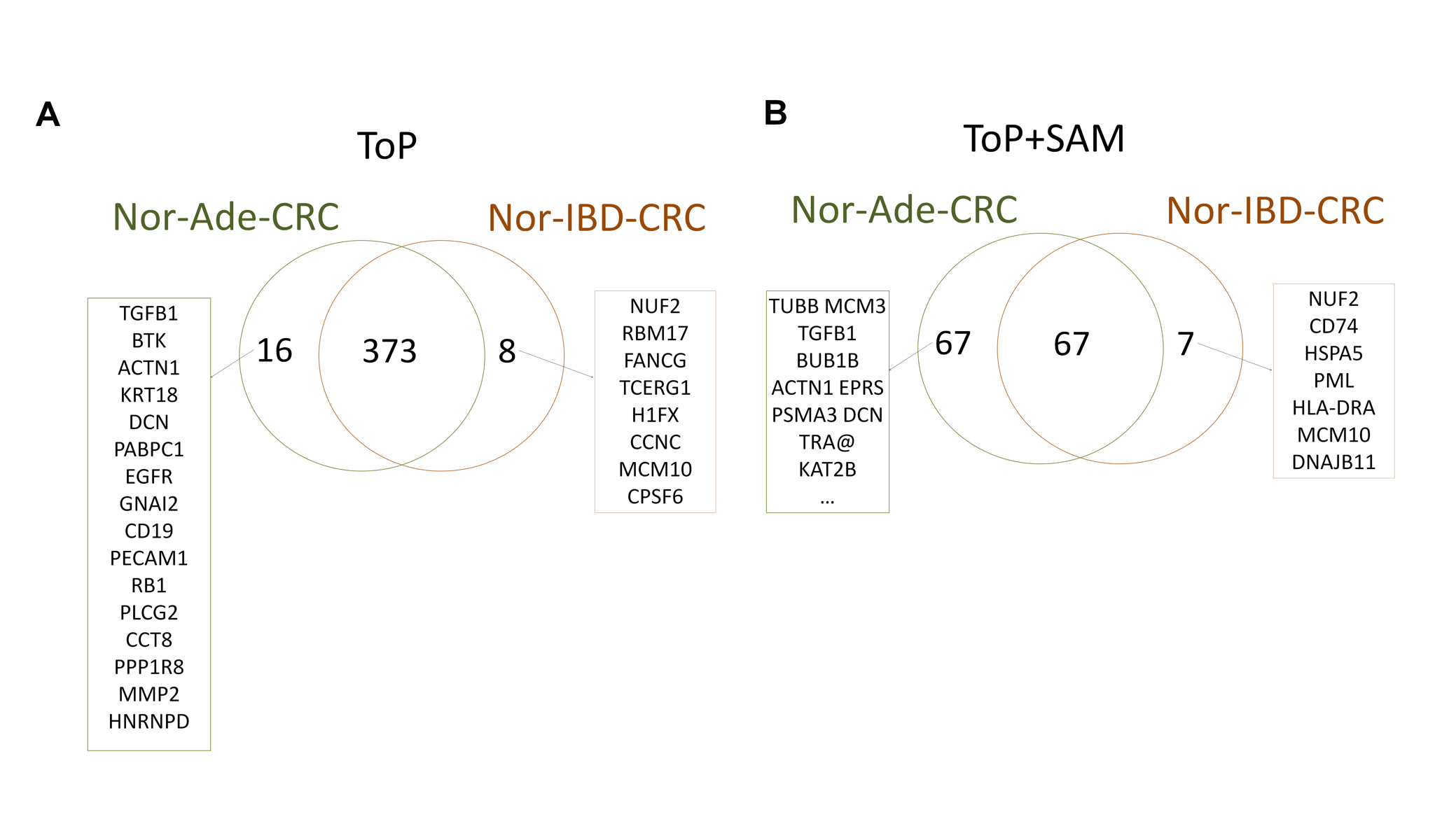

Supplement: Figure S5 — Gene sets selected in the ToP and ToP+SAM (TPS in text) procedures from the Nor-Ade-CRC and Nor-IBD-CRC sequences, and their intersections. (TIF) [file pone.0065683.s005.tif]

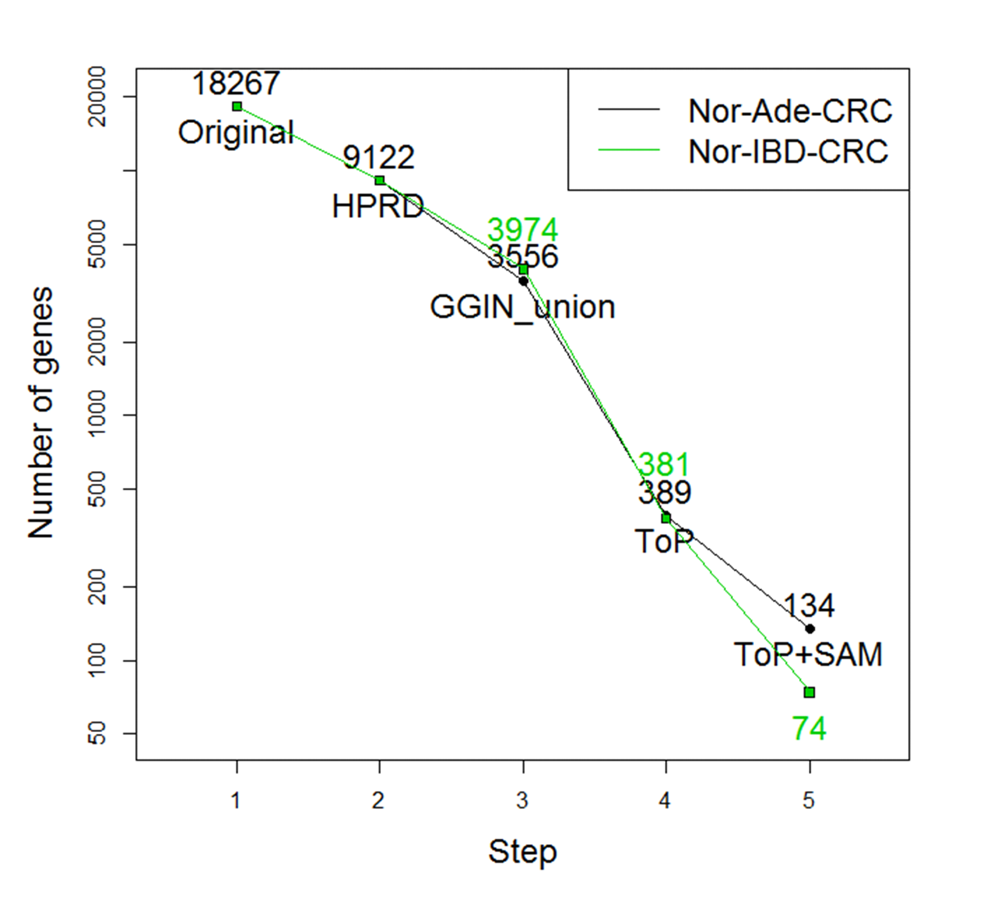

Supplement: Figure S6 — Size of gene set after each stage of screening in the ToP procedure. (TIF) [file pone.0065683.s006.tif]

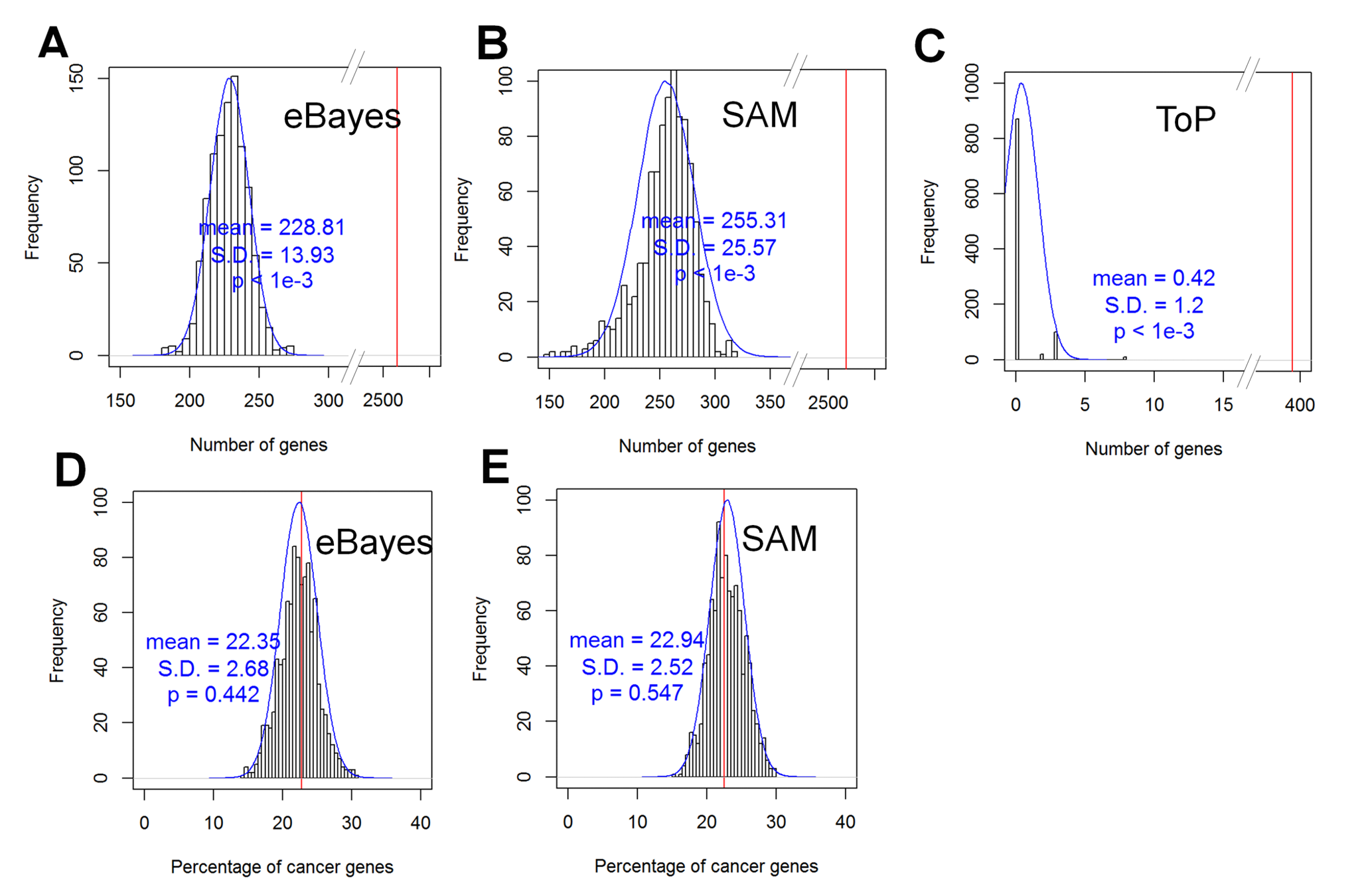

Supplement: Figure S7 — Results from 1000 type-1 randomization tests (see Methods) and in actual cases (red lines). (A–C) Distribution of number of selected genes. (D–E) Distributions of percentages of selected genes listed in CancerGenes [40]. (TIF) [file pone.0065683.s007.tif]

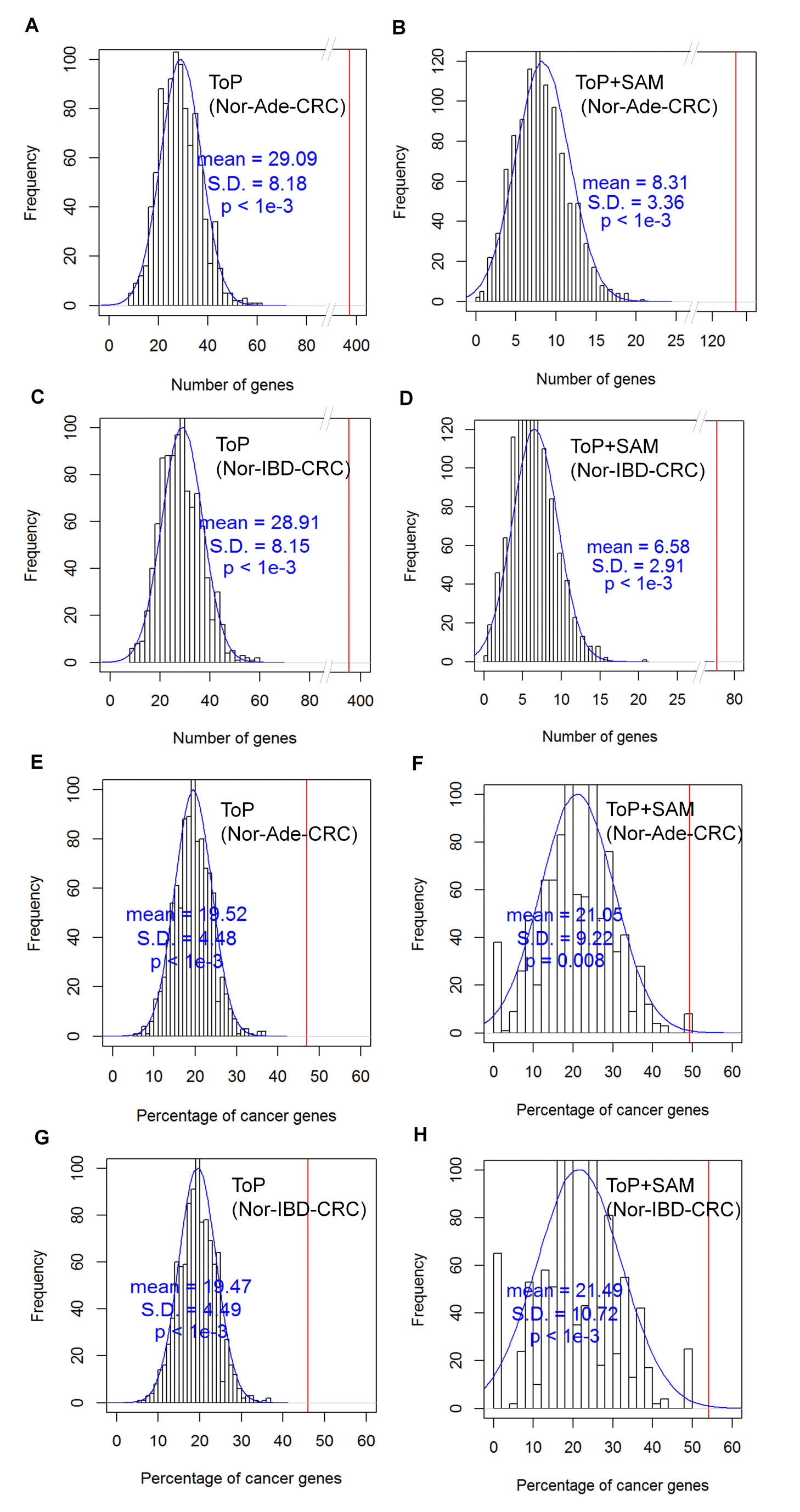

Supplement: Figure S8 — Results from 1000 type-2 randomization tests (see Methods) and in actual cases (red lines). (A–D) Distribution of number of selected genes. (E–H) Distributions of percentages of selected genes listed in CancerGenes [40]. (TIF) [file pone.0065683.s008.tif]

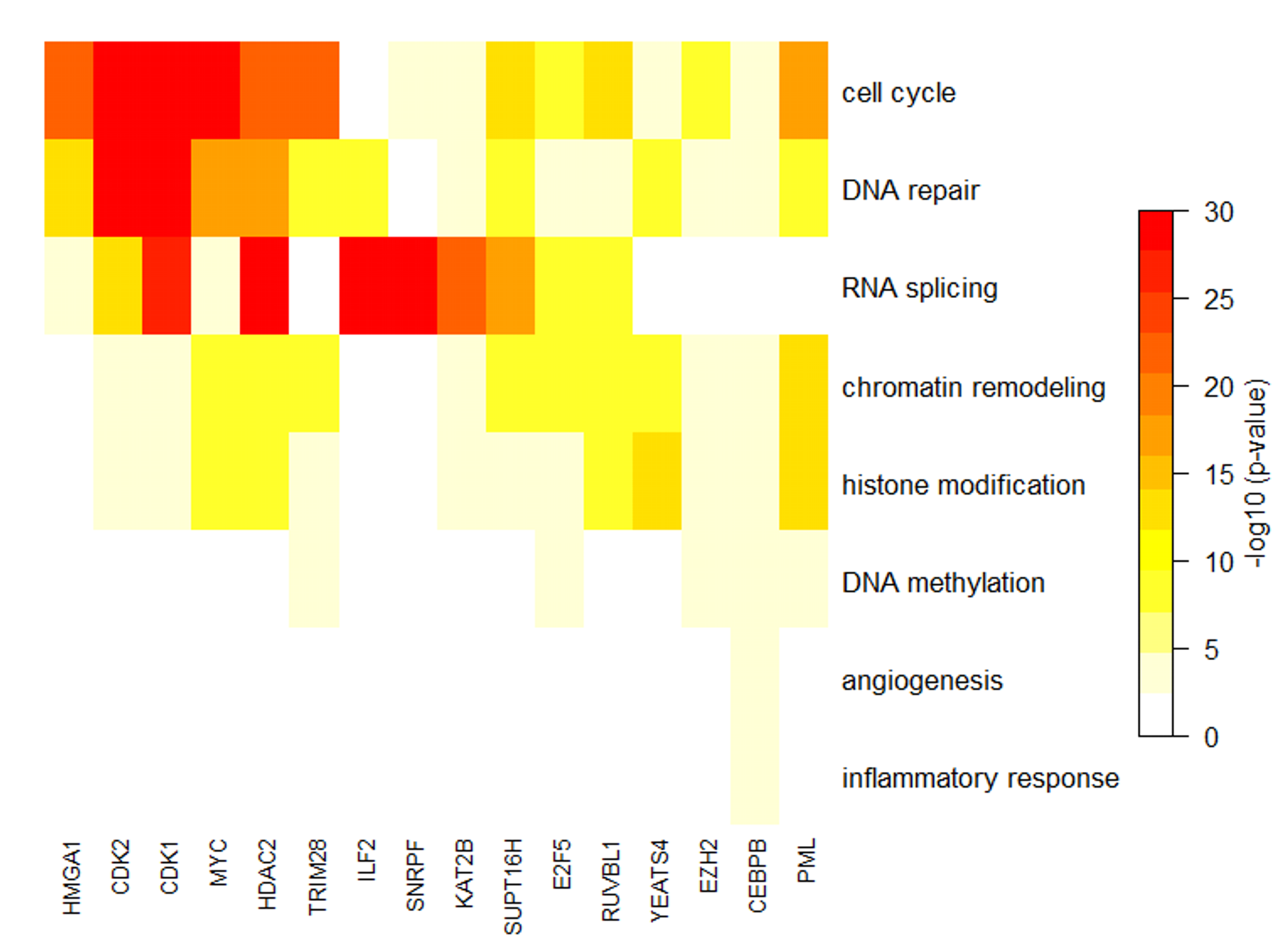

Supplement: Figure S9 — Analysis of gene ontology enrichment in the CRC network of protein modules (right-hand column) regulated by the 16 TFs (bottom) selected by ToP+SAM. (TIF) [file pone.0065683.s009.tif]
